# Impact of a photo intervention on sun safety attitudes

**DOI:** 10.1016/j.jdin.2025.04.004

**Published:** 2025-05-04

**Authors:** Grace Rabinowitz, Benjamin D. Hu, Brandon R. Block, Raphaella Lambert, Hannah Verma, Austin Piontkowski, Camille M. Powers, Jeremy Orloff, Omar Alani, Marianne Tissot, Jesse M. Lewin, Nicholas Gulati, Jonathan Ungar

**Affiliations:** Kimberly and Eric J. Waldman Department of Dermatology, Icahn School of Medicine at Mount Sinai, New York, New York

**Keywords:** behavioral change, patient education, skin cancer prevention, sun protection behavior, sunscreen utilization

*To the Editor:* Skin cancer is the most common type of cancer in the United States,^1^ and surgical excision or destruction are mainstays of treatment.^2^ Visual aids are known to impact behavior change,^3^ but presenting photographs of skin cancer surgeries remains unexplored as an educational intervention.^4^ Compared to graphs and illustrations, photographs are more authentic and may translate to attitudinal change. We conducted a pilot study to understand the effects of these images on attitudes toward sun avoidance using a prospective observational design. Two hundred surveys were completed at an academic dermatology practice in New York City, with 69 completed during total body skin check visits. As a pilot study, power calculations were not performed. The survey response rate was 100%, as all patients determined by dermatologists to be eligible successfully completed the questionnaires. Surveys assessed demographics, skin cancer history, and baseline sun-related behaviors using binary and Likert-scale questions not based on validated questionnaires (Supplementary Methods available via Mendeley at https://data.mendeley.com/datasets/yj6gmrfmmk/2). Images of cancers on the cheek, nose, and ear were presented alongside intraoperative Mohs surgical photos (totaling 6 images). The postintervention survey was administered immediately, assessing attitude rather than behavioral change (Supplementary Tables 1 and II, available via Mendeley at https://data.mendeley.com/datasets/yj6gmrfmmk/2). Patients with prior Mohs surgery were excluded.

Respondents had a mean age of 49.4 and were predominantly female (52%), White (42%), and privately insured (42%) (Supplementary Table III, available via Mendeley at https://data.mendeley.com/datasets/yj6gmrfmmk/2). At baseline, those who had previously discussed the dangers of sun exposure with a physician were more likely to report using sunscreen (*P* = .004). After viewing surgical photos, 66% of respondents reported changing their perception of skin cancer. Based on binary responses, significantly more respondents were motivated to prevent sunspots (65 to 85 respondents, *P* = .005), skin cancer (142 to 162 respondents, *P* = .003), and skin cancer surgeries (71 to 120 respondents, *P* < .001) (Table I). While more respondents were wary of skin aging and sunburn after viewing the photos, these increases were not statistically significant. In addition to increased motivation, respondents reported intentions to significantly change their sun safety behaviors, with more respondents planning to avoid sun, seek shade, as well as wear protective clothing and sunscreen (all *P* < .001).

**Table I:** Changes in motivation and sun-protective behavior

Motivation or sun-protective behavior	Before (*n*)	After (*n*)	*P* value∗
Motivation†			
Prevent skin aging	91	104	.099
Prevent sunburns	77	90	.086
Prevent sunspots	65	85	.005
Prevent skin cancer	142	162	.003
Prevent skin cancer treatment	71	120	<.001
Sun-protective behavior†			
Sun avoidance	76	125	<.001
Seeking shade	113	145	<.001
Wearing protective clothing	58	107	<.001
Wearing a hat	85	123	<.001
Sunscreen use	141	177	<.001
Total (*n*) = 200			

∗ Calculated using McNemar’s test for nonparametric data and Wilcoxon signed rank tests.

† Number of responses who indicated “yes” to each motivation or behavior. Questions were binary (yes/no). An increase in “after” responses indicates a greater number of participants expressing motivation or intention to engage in sun-protective behavior after viewing surgical photos. Participants were asked to select all applicable motivations and behaviors for sun protection.

Of note, responses did not vary based on personal or family history of skin cancer (*n* = 35) (Table II). The lack of motivational differences between these groups highlights the generalizability of our images. However, the magnitude of anticipated behavior change was modest, with planned sunscreen use for an average of 8 months per year after viewing the photos. Limitations include lack of a longitudinal component to assess resilience of attitudinal change.

**Table II:** Sunscreen usage patterns stratified by group before and after photo intervention

Sunscreen usage parameter	Personal/Family history of skin cancer	No personal/Family history of skin cancer	*P* value
Sunscreen use before (%)	29/35 (83)	111/165 (67)	
Planned sunscreen use after (%)	33/35 (94)	143/165 (87)	.381
Months of sunscreen use per year before (mean ± SD)	5.79 ± 4.06	6.66 ± 4.24	
Planned months of sunscreen use per year after (mean ± SD)	7.10 ± 4.39	8.01 ± 4.30	.071
Sunscreen consistent usage before (mean ± SD, scale 1-5∗)	3.24 ± 1.18	3.58 ± 1.27	
Planned consistent usage after (mean ± SD, scale 1-5∗)	4.16 ± 0.99	4.28 ± 1.04	.074

∗ Sunscreen consistent usage measures participants’ subjective assessment of their sunscreen use on a scale from 1 (not consistent use) to 5 (very consistent use). Participants were instructed to rate the consistency of their sunscreen usage without additional guidance regarding specific frequencies.

Viewing images of skin cancer surgeries positively impacted respondents’ motivations to protect themselves from skin cancer and sun exposure, independent of personal or family skin cancer history. While prior physician discussions about sun exposure likely played a role in baseline sunscreen use, this study highlights the unique, independent effect of surgical images in prompting behavioral change. The clinical utility of this tool warrants further investigation in larger populations; nevertheless, our initial findings suggest photos are valuable additions to sun safety counseling techniques.

## Conflicts of interest

None disclosed.
